# Reproduction Function in Male Patients With Bardet Biedl Syndrome

**DOI:** 10.1210/clinem/dgaa551

**Published:** 2020-08-25

**Authors:** Isabelle Koscinski, Manuel Mark, Nadia Messaddeq, Jean Jacques Braun, Catherine Celebi, Jean Muller, Anna Zinetti-Bertschy, Nathalie Goetz, Hélène Dollfus, Sylvie Rossignol

**Affiliations:** 1 Laboratoire de Biologie de la Reproduction/CECOS Lorraine, Hôpitaux universitaires de Nancy, Nancy, France; 2 Université de Lorraine, Inserm, NGERE, Nancy, France; 3 Institut de Génétique et de Biologie Moléculaire et Cellulaire (IGBMC), Illkirch-Graffenstaden, France; 4 Laboratoire de Biologie de la Reproduction, Hôpitaux universitaires de Strasbourg (HUS), Strasbourg, France; 5 Service ORL et CCF, Hôpitaux universitaires de Strasbourg (HUS), Strasbourg, France; 6 Laboratoire de Génétique Médicale, INSERM, UMRS_1112, Institut de Génétique Médicale d’Alsace (IGMA), Fédération de Médecine Translationnelle de Strasbourg (FMTS), Université de Strasbourg, Faculté de médecine de Strasbourg, Strasbourg, France; 7 Laboratoires de Diagnostic Génétique, Hôpitaux Universitaires de Strasbourg, Institut de Génétique Médicale d’Alsace (IGMA), Strasbourg, France; 8 Pôle de Psychiatrie, Santé Mentale et Addictologie, Hôpitaux Universitaires de Strasbourg, Strasbourg, France; 9 Neuropsychologie cognitive et physiopathologie de la schizophrénie, Unité de recherche INSERM U1114, Fédération de Médecine Translationnelle de Strasbourg (FMTS), Université de Strasbourg, Strasbourg, France; 10 Filière SENSGENE, Centre de Référence pour les affections rares en génétique ophtalmologique (CARGO), Institut de Génétique Médicale d’Alsace (IGMA), Hôpitaux Universitaires de Strasbourg, Strasbourg, France; 11 Service de Génétique Médicale, Hôpitaux Universitaires de Strasbourg, Institut de Génétique Médicale d’Alsace (IGMA), Strasbourg, France; 12 Service de Pédiatrie, Hôpitaux Universitaires de Strasbourg, Strasbourg, France

**Keywords:** Bardet Biedl syndrome, male reproduction, male infertility, primitive ciliopathy, genetic

## Abstract

**Purpose:**

Bardet-Biedl syndrome (BBS) is a ciliopathy with a wide spectrum of symptoms due to primary cilia dysfunction, including genitourinary developmental anomalies as well as impaired reproduction, particularly in males. Primary cilia are known to be required at the following steps of reproduction function: (i) genitourinary organogenesis, (ii) in fetal firing of hypothalamo-pituitary axe, (iii) sperm flagellum structure, and (iv) first zygotic mitosis conducted by proximal sperm centriole. BBS phenotype is not fully understood.

**Methods:**

This study explored all steps of reproduction in 11 French male patients with identified BBS mutations.

**Results:**

BBS patients frequently presented with genitourinary malformations, such as cryptorchidism (5/11), short scrotum (5/8), and micropenis (5/8), but unexpectedly, with normal testis size (7/8). Ultrasonography highlighted epididymal cysts or agenesis of one seminal vesicle in some cases. Sexual hormones levels were normal in all patients except one. Sperm numeration was normal in 8 out of the 10 obtained samples. Five to 45% of sperm presented a progressive motility. Electron microscopy analysis of spermatozoa did not reveal any homogeneous abnormality. Moreover, a psychological approach pointed to a decreased self-confidence linked to blindness and obesity explaining why so few BBS patients express a child wish.

**Conclusions:**

Primary cilia dysfunction in BBS impacts the embryology of the male genital tract, especially epididymis, penis, and scrotum through an insufficient fetal androgen production. However, in adults, sperm structure does not seem to be impacted. These results should be confirmed in a greater BBS patient cohort, focusing on fertility.

The multisystem involvement of the Bardet-Biedl syndrome (BBS, OMIM 209900) is related to the pathogenesis well recognized as a ciliopathy. Its estimated prevalence in North America and Europe ranges from 1:140 000 to 1:160 000 live births (1). In the last 20 years, 22 genes have been implicated in BBS (2, 3). All of them participate in primary cilia function by coding for proteins involved in the formation of the BBSome (4), the chaperonin complex (5), or the basal body, with an essential role in the intraflagellar traffic (6, 7).

BBS phenotype is characterized by retinal dystrophy, obesity, renal dysfunction, learning difficulties, genital anomalies, and postaxial polydactyly (a pathognomonic sign in this context). Hypogonadism is part of the major diagnosis criteria in males (8, 9). The generic term of hypogenitalism is often used in clinical series and micropenis, cryptorchidism, and delayed puberty are frequently reported (8, 10). However, hormonal and histological data are scarce and the origin of this hypogonadism (primary or hypogonadotropic) remains unclear. Large BBS series stipulate that males are almost invariably infertile (9) and only a few males with descendants are reported (8). By way of a confused extrapolation to the Kartagener syndrome, another ciliopathy (affecting motile cilia), BBS patients are supposed to be infertile by producing immotile spermatozoa (9).

Moreover, the potential impact of primary cilia on fertility is considered to result from the role of cilia structures at different steps of human reproduction function and from a partial common ultrastructural architecture of all cilia (11).

The present study explores the reproduction function in a cohort of BBS male patients with identified mutations within different BBS genes. The results are interpreted in the context of organogenesis of male genital organs, hormonal secretions of the hypothalamic-pituitary-gonadal axis, spermatogenesis, and sexual/reproductive behavior. To our knowledge, this is the first specific study of the reproduction function of BBS male patients, contrasting with the presumption that BBS patients are infertile.

## Subjects and Methods

Subjects

Subjects were recruited by the reference center for rare eye diseases at the Strasbourg University Hospital, France (CARGO) and were explored for fertility by the Centre of Medicine and Biology of Reproduction at the Strasbourg University Hospital, France. In total, 11 adult male BBS patients underwent a complete exploration of the gonadotropic axis including clinical examination, comprehensive male hormonal panel testing, including a gonadotropin-releasing hormone (GnRH) stimulation test (0.1 mg Relefact; Sanofi-Aventis, Frankfurt am Main, Germany), urinary-genital ultrasonography, standard sperm analysis with a modified David classification of morphological anomalies (12), and TEM whenever sperm count was high enough. The clinical examination comprised: (i) palpation of testes to evaluate scrotum length and testes volume; (ii) examination of the penis to evaluate the presence of micropenis (when the length of flaccid penis was less than 2.5 SD—measurement taken from the pubic ramus to the distal tip of the gland) (13-15); (3) evaluation of the secondary sexual characteristics; and (4) presence of gynecomastia. This exploration was a part of the French National Research Protocol ethically approved by CPP “EST IV” (Strasbourg, France) (PHRC National Bardet-Biedl 2007 IDRCB 2007-A00868-45); it included also ophthalmic, olfactive, endocrine, and psychological explorations. Furthermore, 1 patient consulted with his wife for infertility and underwent an assisted reproductive technology (ART) program comprising 3 intrauterine artificial inseminations and one in vitro fertilization (IVF) assisted by intracytoplasmic sperm injection (ICSI).

## Mutation Analysis

Genomic DNA was isolated and explored as previously described either by direct Sanger sequencing or by high-throughput sequencing (16).

### Sperm analysis

All sperm analyses were performed in accordance with the World Health Organization recommendations (17); the morphology was analyzed using David modified criteria (18).

### Transmission electron microscopy (TEM)

Spermatozoa were fixed at 4 °C in 2.5% glutaraldehyde for 2 hours, then centrifuged. The pellet was washed in sodium cacodylate buffer (0.1 M), then postfixed in 1% osmium tetroxide in cacodylate buffer and washed again. After a progressive dehydration in ethanol (50°-70°-95°-absolute [3 times]), propylene oxide (3 times), each step 10 minutes at room temperature, the pellet was embedded in epoxy (Epon) resin and ultrathin sections were obtained.

### Olfactory evaluation

 A senior ear nose throat (ENT) specialist evaluated all BBS patients according to the same protocol as described in Braun et al (19): (i) clinical evaluation of olfaction, (ii) ENT examination with nasal endoscopy and (iii) olfactometry using 2 different psychophysical methods, namely a suprathreshold evaluation of the olfaction and the UPSIT (Sensonics Inc., Haddon Heights, NJ) (20-22).

### Verbal intelligence quotient evaluation

As described in Braun et al (19), the estimated Verbal Intelligence Quotient (VIQ) was calculated on the basis of the administration of subscales from the Wechsler Adult Intelligence Scale (WAIS-III) (23).

## Results

The cohort analyzed in this study was composed of 11 male patients with a clear clinical and molecular diagnosis of BBS. Patients were from 18 to 39 years of age. The main elements of their medical history, clinical examination, and genotype are summarized in Table 1 (24, 25). All patients of this cohort presented primary and secondary recognized features of BBS: all presented with rod-cone dystrophy. Polydactyly or brachydactyly was observed for 9 of the 11. Similarly, 8 out of the 11 patients were obese and 3 of them had morbid obesity. Moreover, 2 patients had hyperinsulinism with glucose intolerance and 1 was diabetic. Interestingly, 7 out of 10 patients evaluated for olfaction presented an anosmia or severe hyposmia (19).

**Table 1.** Clinical and Genetic Data for the Cohort

Patient	Age (years)	Mutation	Clinical and Medical Background								
			Rod-cone dystrophy	Hands/feet anomalies	BMI	Micropenis	Cryptorchidism	Kidney cysts or ectasia	Diabetes, glucose intolerance	Olfaction defect	VIQ
**1**	**22**	*BBS1*: c.[479G>A];[479G>A], p.[Arg160Gln];[Arg160Gln]	yes	brachydactyly	>30	NA	no	no	no	mild hyposmia	60.5
**2**	**25**	*BBS1*: c.[1169T>G];[1169T>G], p.[Met390Arg];[Met390Arg]	yes	no	<30	no	no	no	no	normal	64
**3**	**24**	*BBS1*: c.[1169T>G];[1169T>G], p.[Met390Arg];[Met390Arg]	yes	no	<30	no	right	no	no	severe hyposmia	62
**4**	**36**	*BBS1*: c.[382C>T];[382C>T], p.[Gln128*];[Gln128*]	yes	polydactyly	>30	yes	bilateral	no	no	NA	NA
**5**	**24**	*BBS5*: c.[413G>C];[413G>C], p.[Arg138Pro];[Arg138Pro]	yes	polydactyly	>30	yes moderate	right	yes	no	severe hyposmia	93
**6**	**35**	*BBS10*: c.[271dup];[963T>G], p.[Cys91Leufs*5];[Tyr321*]	yes	polydactyly brachydactyly	>40	yes	bilateral	yes	no	anosmia	90
**7**	**38**	*BBS1*: c.[1169T>G];[1214_1215ins[MT113356]], p.[Met390Arg];[(Ala406Glnfs*47)]	yes	polydactyly	<25	NA	no	yes	no	severe hyposmia	62
**8**	**22**	*BBS1*: c.[1169T>G];[1169T>G], p.[Met390Arg];[Met390Arg]	yes	polydactyly	>40	yes	no	yes	no	moderate hyposmia	94.5
**9**	**26**	*BBS9*: c.[703-?_886+?del];[832C>T], p.[Val235Phefs*6];[Arg278*]	yes	polydactyly	>40	no but angulation	no	yes, mild form	yes	anosmia	102
**10**	**18**	*BBS3*: c.[535G>A];[535G>A], p.[Asp179Asn];[Asp179Asn]	yes	polydactyly	>30	NA	no	yes	yes	anosmia	88.5
**11**	**39**	*BBS12*: c.[1037T>C];[1037T>C], p.[Ile346Thr];[Ile346Thr]	yes	polydactyly	>35	yes	left	yes	yes	anosmia	68.5
**Total patients**	**Median 25**		11/11	9/11	8/11	5/8	5/11	7/11	3/11	9/10	Median 78.5

Mutations in patient 1 and 10 affect the splicing of their respective gene (24, 25). Abbreviations: NA, not available; VIQ, Verbal Intelligence Quotient.

Genital examination and hormonal status assessment were part of the protocol; however, 3 patients refused external genital clinical examination. Andrological data are summarized in Table 2 and hormonal status in Table 3: BBS patients frequently presented with a history of nondescended testis (5 out of 11; 3 unilateral and 2 bilateral) and/or a short scrotum at adult age (5 out of 8). The length of penis was much decreased (<−2.5 SD) in 5 out of the 8 examined patients, 3 had a normal length, and 1 presented an abnormally curved penis. Unexpectedly, the size of the testes was normal (7 out of 8). They all reported a normal spontaneous puberty. The systemic androgenic effects, evaluated by the general body hair and sexual androgen dependant body hair, was normal in 7 out of 8 examined patients.

**Table 2.** Andrological Data

Patient	Age (years)	Mutation	Medical background and andrological examination				Genito-urinary ultrasonography	Kidney ultersonography (kidney cysts or ectasia)
			Cryptorchidism	micropenis	testis size	scrotum length		
**1**	**22**	BBS1: c.[479G>A];[479G>A], p.[Arg160Gln];[Arg160Gln]	no	Examination not performed	Examination not performed	Examination not performed	normal	no
**2**	**25**	BBS1: c.[1169T>G];[1169T>G], p.[Met390Arg];[Met390Arg]	no	no	normal	normal	not performed	no
**3**	**24**	BBS1: c.[1169T>G];[1169T>G], p.[Met390Arg];[Met390Arg]	right	no	normal	normal	not performed	no
**4**	**36**	BBS1: c.[382C>T];[382C>T], p.[Gln128*];[Gln128*]	bilateral	yes	Bilateral mild hypotrophy	short	right seminal vesicle non observed; right epididymal cyst	no
**5**	**24**	BBS5: c.[413G>C];[413G>C], p.[Arg138Pro];[Arg138Pro]	right	yes moderate	normal	short	right epididymal cyst	yes
**6**	**35**	BBS10: c.[271dup];[963T>G], p.[Cys91Leufs*5];[Tyr321*]	bilateral	yes	normal right, mild hypotrophy of left testis associated with varicocele; bilateral epididymal cysts	normal	right seminal vesicle non observed; left varicocele; bilateral epididymal cysts	yes
**7**	**38**	BBS1: c.[1169T>G]; [1214_1215ins[MT113356], p.[Met390Arg];[Ala406Glnfs*47]	no	not performed	not performed	not performed	not performed	yes
**8**	**22**	BBS1: c.[1169T>G];[1169T>G], p.[Met390Arg];[Met390Arg]	no	yes	normal	very short	enlargement of prostatic utricle	yes
**9**	**26**	*BBS9*: c.[703-?_886+?del];[832C>T], p.[Val235Phefs*6];[Arg278*]	no	no but angulation	normal	short	not performed	yes mild form
**10**	**18**	BBS3: c.[535G>A];[535G>A], p.[Asp179Asn];[Asp179Asn]	no	not performed	not performed	not performed	not performed	yes
**11**	**39**	BBS12: c.[1037T>C];[1037T>C], p.[Ile346Thr];[Ile346Thr]	left	yes	bilateral mild hypotrophy	short	not performed	yes

**Table 3.** Hormonal Status

Patient	Age (years)	Mutation	Hormonal *levels*							
			Basal FSH IU/L [1.5-12]	FSH at the peak (after GnRH stimulation) [1.5–2 × basal]	Basal LH IUI/L [1.5-8.5]	LH at the peak (after GnRH stimulation) [3-4 × basal]	Basal total testosterone nmol/L [10.4-41.6]	Testosterone/LH (nMol / IU)	PRL mIU/L [86-324]	Leptin (µg/L)
**1**	**22**	BBS1: c.[479G>A];[479G>A], p.[Arg160Gln];[Arg160Gln]	2.42	4.65	4.71	29.3	16.3	3.46	239	21.2
**2**	**25**	BBS1: c.[1169T>G];[1169T>G], p.[Met390Arg];[Met390Arg]	2.67	5.02	2.94	20.8	not performed		not performed	8.83
**3**	**24**	BBS1: c.[1169T>G];[1169T>G], p.[Met390Arg];[Met390Arg]	5.37	7.15	3.21	15.7	17.7	5.51	176	not performed
**4**	**36**	BBS1: c.[382C>T];[382C>T], p.[Gln128*];[Gln128*]	not performed	not performed	not performed	not performed	not performed		not performed	not performed
**5**	**24**	BBS5: c.[413G>C];[413G>C], p.[Arg138Pro];[Arg138Pro]	3.26	5.94	3.64	20.6	13.8	3.79	134	12.6
**6**	**35**	BBS10: c.[271dup];[963T>G], p.[Cys91Leufs*5];[Tyr321*]	2.08	3.69	3.38	12.5	12.8	3.77	106	not performed
**7**	**38**	BBS1: c.[1169T>G]; [1214_1215ins[MT113356], p.[Met390Arg];[Ala406Glnfs*47].	3.51	6.37	4.11	26.9	11.1	2.70	94.7	4.78
8*	**22**	BBS1: c.[1169T>G];[1169T>G], p.[Met390Arg];[Met390Arg]	0.85	not performed	0.71	not performed	18.7*	Not applicable	295	19.8
**9**	**26**	BBS9: c.[703-?_886+?del];[832C>T], p.[Val235Phefs*6];[Arg278*]	1.56	3.16	1.66	12	6.9	4.15	normal	50.4
**10**	**18**	BBS3: c.[535G>A];[535G>A], p.[Asp179Asn];[Asp179Asn]	2.84	8.46	3.57	51.8	8.6	2.4	72.1	not performed
**11**	**39**	BBS12: c.[1037T>C];[1037T>C], p.[Ile346Thr];[Ile346Thr]	9.41	20.5	7.02	43	11.1	1.58	209	not performed

*Patient 8 undergoing testosterone therapy

Genito-urinary ultrasonography was performed in 5 patients and highlighted abnormalities, such as a cyst of prostatic utricle (1 case), epididymal cysts (1 case), or unilateral agenesis of seminal vesicle (3 out of 5 cases), that could be related to extreme hypospermia (<0.5 mL in 2 cases). Kidney cysts or ectasia were found in 7 out of 11 patients (2 with normal semen volume, 4 with decreased semen volume, and 1 patient with failure of semen collection).

Testosterone and gonadotropin levels (Table 3) were not interpretable in patient 8, since he was undergoing testosterone therapy: he was then considered as having a hypogonadism (previous hormonal status not available). For the other patients, testosterone was low only in 2 cases out of 8 (2 missing data) and basal gonadotrophins were all in normal range (1 missing data). For all tested patients, the pituitary response to a GnRH stimulation test was normal (9/9). The testosterone/luteinizing hormone (LH) ratio was calculated for 8 out of 11 patients and ranged from 1.58 to 5.51.

**Figure 1.**
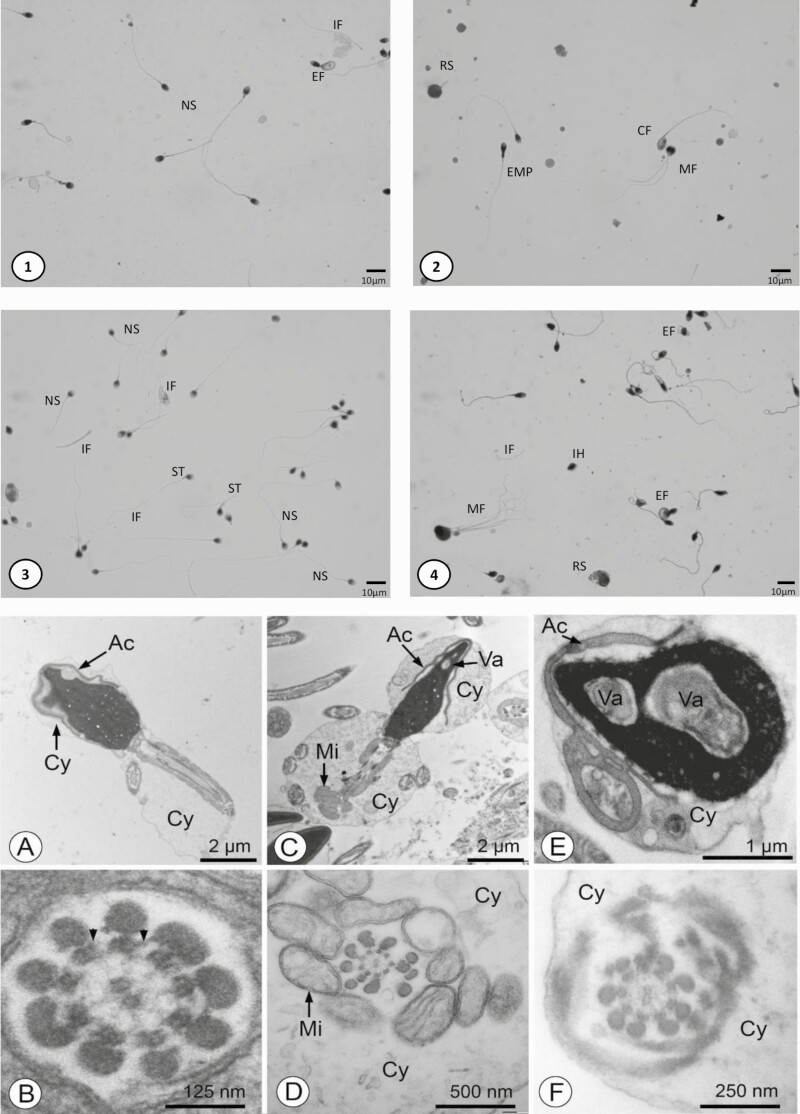
Sperm morphology in BBS patients. Panels 1-4: Harris Shorr staining, from different patients, (1) Patient 2; (2) Patient 8; (3) Patient 6; (4) Patient 9. Abbreviations: CF, coiled flagellum; EMP, enlarged middle piece; IF, isolated flagellum; IH, isolated head; MF, multiflagellar sperm; NS, normal spermatozoa; RS, round spermatid; ST, short tail. The proportion of isolated flagella is increased. Coiled flagellum, short tail, enlarged middle piece suggest an impaired spermiogenesis. Panels A-F. TEM analysis of spermatozoa. (A and B) and (C-F) correspond to 2 different patients. (A, C-F) Commonly observed defects include: acrosomes (Ac) detached from the nucleus (in A, C, E) and malformed (in E), excess of cytoplasm (Cy) in the head (in A, C, E) and flagellum (in D, F), disorganized mitochondrial sheaths (Mi) (in C, D) and large nuclear vacuoles (Va) (in C, E). (B, D, F) The majority of the cross sections of axonemes show a normal 9 plus 2 microtubule pattern and well-preserved dynein arms (arrowheads in B). Note that abnormalities of axonemes in the spermatozoa (eg, absence of the central pair of microtubules; not illustrated here) are almost systematically associated with poor preservation of the adjacent mitochondria, therefore strongly suggesting that they reflect a state of cellular necrosis.

Sperm analysis was performed in 10 of the 11 patients (Table 4) as 1 failed to collect semen (only 0.12 mL of glandular secretion without epididymal fraction of ejaculate). A decreased semen volume was observed in 6 out of the 10 patients (median of 1.2 mL (0.1, 12.8), and a very low semen volume (<0.5 mL) was observed in 3 out of 10 patients performing a semen collection. Sperm numeration was normal in 8 out of 10 patients (median of 72 × 10^6^ [0, 485 × 10^6^]), and sperm progressive motility ranged from 5% to 45% (median of 23%). The teratozoospermia was moderately increased (median 8% of typical forms [2, 28]), and abnormalities were heterogeneous. These findings were confirmed by TEM analysis: more than 80% of the spermatozoa displayed severe morphological alterations of the head (eg, large nuclear vacuoles) and/or the midpiece (eg, disorganized mitochondrial sheath), which were apparently combined at random. TEM analysis also revealed that the normal microtubule pattern of the axoneme, with nine doublets surrounding a pair of singlets was preserved in most of transverse sections through the midpiece or principal piece. Therefore, these spermatozoa were without any specific anomaly of the axoneme, having notably a central pair of microtubules as well as para-microtubular proteins like dynein arms (Fig. 1).

**Table 4.** Sperm Analysis

Patient	Age (years)	Semen volume (mL)	pH	Viscosity	Numeration spz/Ejaculate (N > 39 × 10^6^)	Progressive motility (%) (N > 33%)	Total motility (%) (N > 50%)	Vitality (%) (N > 58%)	Morphology according to David modified criteria (N TF > 12%)
**1**	**22**	12.8	6	normal	0.09 × 10^6^	0	0	Not performed	10/19 enrolled tail, 18/19 abnormal acrosome, 1/20 TF
**2**	**25**	1.2 (da = 2j)	8.1	Normal	324 × 10^6^	45	50	69	6% enrolled tail, 68% abnormal acrosome; 11% TF; isolated tails: 17% of observed spermatozoa
**3**	**24**	2.3	7.8	Normal	138 × 10^6^	25	30	61	32% enrolled tail, 86% abnormal acrosome, 2% TF; isolated tails: 85% of observed spermatozoa
**4**	**36**	6.5	6.8	Normal	15	0	0	0	not interpretable
5*	**24**	0.12 *	6.3	Normal	0	Not applicable	Not applicable	Not applicable	not applicable
**6**	**35**	0.3	7.8	Normal	79.2 × 10^6^	37	52	>60	50% abnormal tail, 11 à 20% TF
**7**	**38**	0.3	8.1	Normal	217 × 10^6^	20	30	>30	28% TF
**8**	**22**	2.7	6.6	Normal	32.1 × 10^6^	9	11	33	13% multitailed sperm; 14%enrolled tail; 15% isolated tails; 4% TF
**9**	**26**	0.9 (DA = 2j)	7.5	Normal	62.3 × 10^6^	5	10	66	8% TF; 6% isolated tails
**10**	**18**	2.1	7.5	Normal	481 × 10^6^	26	35	60	39% abnormal tail; 8%TF; <5% isolated tails
**11**	**39**	0.45	8.1	normal	72 × 10^6^	26	31	48	7% TF 82% abnormal acrosome; 12% isolated tails

Abbreviations: spz, spermatozoa; TF, typical form.

*Patient 5 failed to produce ejaculate.

The *BBS1* gene was implicated in 6 out of the 11 cases. Other cases comprised homozygous mutation in *BBS3/ARL6, BBS5, BBS9, BBS12* or compound heterozygous mutations in *BBS10*. Regarding the reproductive function, no genotype to phenotype association could be observed in our cohort. Even between siblings, variations of the phenotype were observed: only 1 of the siblings, with the same homozygote mutation of BBS1 (c.[1169T>G];[1169T>G], p.[Met390Arg];[Met390Arg]), reported a cryptorchidism history. Another patient bearing the same mutation presented a severe decrease of semen volume (0.3 mL).

The psychological evaluation of patients (Tables 1 and 5) uncovered a moderate mental retardation with a Verbal Intellectual Quotient varying from 60.5 to 102 (median 78.5) associated with a frequent lack of self-confidence mainly due to their obesity. Two patients lived with a partner; only 1 consulted for infertility and the couple underwent ART.

**Table 5.** Psychological Concerns

Patient	Age (years)	Mutation	Clinical data			Lifestyle		
			BMI	blindness	VIQ	Profession	Living with a partner	Child wish
**1**	**22**	BBS1: c.[479G>A];[479G>A], p.[Arg160Gln];[Arg160Gln]	>30	yes	60.5	baker	no	no
**2**	**25**	BBS1: c.[1169T>G];[1169T>G], p.[Met390Arg];[Met390Arg]	<30	yes	64	telephone advisor	no	no
**3**	**24**	BBS1: c.[1169T>G];[1169T>G], p.[Met390Arg];[Met390Arg]	<30	No, residual visual acuity	62	telephone advisor	yes	yes
**4**	**36**	BBS1: c.[382C>T];[382C>T], p.[Gln128*];[Gln128*]	>30	yes	not performed	metallurgy worker	yes	no
**5**	**24**	BBS5: c.[413G>C];[413G>C], p.[Arg138Pro];[Arg138Pro]	>30	yes	93	groom	no	no
**6**	**35**	BBS10: c.[271dup];[963T>G], p.[Cys91Leufs*5];[Tyr321*]	>40	yes	90	office worker	no	no
**7**	**38**	*BBS1*: c.[1169T>G];[1214 _1215ins[MT113356]], p.[Met390Arg];[(Ala406 Glnfs*47)]	<25	yes	62	professionnal sportman	no	no
**8**	**22**	BBS1: c.[1169T>G];[1169T>G], p.[Met390Arg];[Met390Arg]	>40	yes	94.5	economy student	no	no
**9**	**26**	*BBS9*: c.[703-?_886+?del];[832C>T], p.[Val235Phefs*6];[Arg278*]	>40	yes	102	telephone advisor	no	no
**10**	**18**	BBS3: c.[535G>A];[535G>A], p.[Asp179Asn];[Asp179Asn]	>30	yes	88.5	musician (guitarist)	no	no
**11**	**39**	BBS12: c.[1037T>C];[1037T>C], p.[Ile346Thr];[Ile346Thr]	>35	yes	68.5	wood sawmill worker	no	no

## Discussion

Among the main phenotypic features of BBS, hypogenitalism/hypogonadism is often used as a generic term, encompassing cryptorchidism, short scrotum, micropenis, and low testicular volume that also has suggested associated infertility (9). If the literature frequently reports genital anomalies (8-10, 26), in contrast, hormonal assessment is rarely performed. In some cases of BBS, the presence of hypogonadotropic hypogonadism was concluded (27-29), while in others, the presence of hypogonadism of testicular origin (30-33), or both mechanisms were reported (34). Of note, in these reports, the diagnosis of BBS was only clinically assessed, and some patients were prepubertal or young adolescents.

In order to document this aspect of the syndrome, we have systematically analyzed the reproductive function of 11 male BBS patients. As illustrated in Tables 2, 3, and 4, BBS patients present an important variability of features (clinical and biological) linked to fertility.

Five patients had a history of bilateral (n = 2) or unilateral cryptorchidism and 4 of them exhibited a micropenis and short scrotum. This association strongly suggests a congenital hypogonadotropic hypogonadism (CHH) (35, 36) with insufficient hypothalamic-pituitary–induced androgen secretion after midgestation that is normally responsible for inguino-scrotal testes descent and penile growth. The neuro-endocrine regulation of GnRH hypothalamic release is quite complex and results from the interplay of activating and inhibitory inputs to GnRH neurons during fetal life. The recent description of the fundamental role of hypothalamic kisspeptin and its receptor Kiss1r (previously named GPR54) (37) on GnRH secretion brought a major advance in the understanding in this field (for an extensive review, see (37)). The regulation of GnRH release by kisspeptin signaling appears crucial for the functional integrity of the gonadotropic axis during fetal life, for pubertal onset, and to maintain fertility in adults (37, 38). Interestingly, in the mouse brain, primary cilia of GnRH neurons are enriched in Kiss1r and Kiss1r activity, in response to kisspeptin binding, is enhanced by the presence of cilia on GnRH neurons (39). This signal amplification appears determinant in the firing rate of fetal GnRH neurons, especially in males. Koemeter-Cox et al (39) also showed that the percentage of GnRH neurons displaying at least 1 Kiss1r-positive cilium was 75% in both sexes at birth and was stable throughout the lifetime. However, quantifying the percentage of GnRH neurons possessing more than 1 Kiss1r positive cilium revealed that the frequency of multiciliate GnRH neurons significantly increases during postnatal development (from 10% at birth to 42% at P60) in parallel with sexual maturation. In humans, as well as in rodents, signaling in the GnRH neurons is the result of clustering of receptors in the ciliary membrane (11, 39). Any dysfunction of primitive cilia in BBS patients could explain a decreased activity of KISS1R signaling pathway during fetal and early postnatal life leading to micropenis and undescended testis.

This CHH seems reversible in most cases, since all patients presented a spontaneous puberty, normal secondary sexual characteristics, and normal testicular volume. Testosterone levels at adult age were indeed normal in all except 2 patients with relatively low testosterone and 1 who had been undergoing testosterone replacement for years. Reversal of CHH occurs in 10% to 20% of patients, which challenges the dogma that the condition is lifelong (35). Desai et al have described a case of reversible hypogonadotropic hypogonadism in a BBS man (40). We propose the hypothesis that in BBS patients, the increase of cilia number on GnRH neurons during puberty, as observed in mice, leading to an increase in KISS1Rs number, might overcome the individual signal reduction and allow a normal puberty and normal adult gonadotropic axis in most of cases. Many case reports focused on pubertal delay (32, 34, 40). Unfortunately, exact timing and tempo of puberty could not be assessed in our patients since it was a retrospective declarative data. Following along the lifespan testicular function in these patients could be of interest, since cryptorchidism and obesity can both impact on gonadal function, as suggested by the lowest ratio of testosterone/LH observed in the oldest patient of our series.

**Figure 2.**
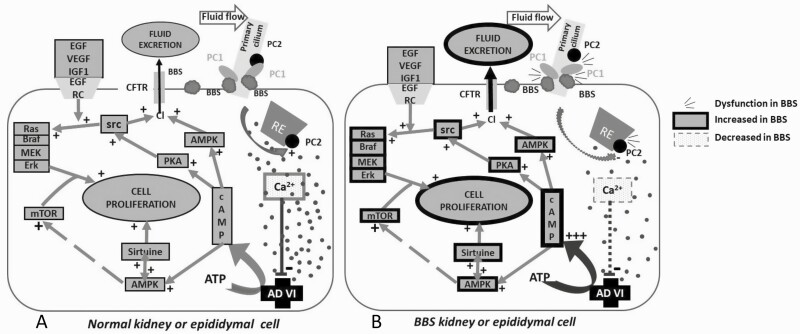
Main mechanisms involved in the cystogenesis. A: In epithelial kidney or epididymal cells, primary cilia constitute a reserve of polycystin 1 (PC1) and polycystin 2 (PC2). PC1 assembly with PC2 to form a dimer inducing from the endoplasmic reticulum an exit of calcium. A high concentration of cytoplasmic Ca2+ represses adenylate cyclase VI (AC VI). The AC VI produces cAMP stimulating AMPk pathway and PKA pathway which are resulting in a stimulation of cell proliferation and CFTR-linked chloride secretion respectively (42). BBSome of the primary cilia stabilizes the PC1 complex and allows a functional dimerization with PC2 (43). B: An anomaly of BBSome decreases the stability of PC1-PC2 dimer (43), resulting further in an insufficient Ca2+ intracytoplasmic concentration, an insufficient repression of AC VI and finally to an excessive cell proliferation and excretion of chloride and fluid through the CFTR. Excessive cell proliferation and excessive fluid secretion lead to cyst formation.

The frequent occurrence of cystic formation in male genital tract, seminal vesicles and kidneys can be explained by the fact that epithelial cells in these structures share a similar embryonic origin from the intermediate mesoderm and expression of primitive cilia. The high rate of kidney cysts observed in BBS patients is in favor of this hypothesis (7 out of 11 in our series, in accordance with the literature (16)). We hypothesize that cyst formation in the male genital tract of BBS patients results from a physio-pathological mechanism similar to that described in polycystic kidney disease: cyst formation would result from a dysfunction of the dimer polycystin 1–polycystin 2 (PC1-PC2) (11, 41, 42), inducing excess cell growth, proliferation, and secretion. In accordance with a recent discussion about the role of BBS proteins in the cystogenesis (43), we presume that, in ciliated epididymal cells like in kidney cells, BBSome interacts with PC1 to stabilize the complex (Fig. 2). Expression of a pathogenic BBS3/Arl6 mutant (T31R) locks Arl6 in the GDP form leading to stunted cilia and inhibition of PC1 on primary cilia (44).

The diminution of semen volume appears to be due more to a partial obstruction of the genital duct by cysts of the genital tract, rather than to hypogonadotropic hypogonadism not observed in our series. In patient 8, ultrasonography revealed the presence of a cyst of prostatic utricle, which is an embryologic Müllerian remnant and should not be confused with other ciliopathy-related cysts of the genital tract, which could also affect the prostate. Another cause of hypovolemia is the unilateral agenesis of a seminal vesicle, potentially related to the prenatal embryogenesis of this gland (45, 46), which begins by GW 14 to 16 from the Wolffian duct and is dependent on testosterone. The prenatal defect of fetal testosterone production linked to prenatal hypogonadotropic hypogonadism in BBS patients could explain this rather frequent abnormality (2 patients out of 5 patients explored by ultrasonography).

Furthermore, this study discloses the conserved motility of the spermatozoa in a great proportion of the BBS patients studied as the integrity of the 9 peripheral + 1 central pair of microtubules is conserved. The variable asthenozoospermia observed (5%-45% of progressive motility) in the series could rather be due to the poor spermatogenesis conditions due to the short scrotum and the obesity disturbing scrotal thermoregulation (47). The notion of infertility in BBS patients linked to a suspected major asthenozoospermia results from a confusion between motile cilia and primary cilia. Conserved sperm motility as well as results of ART performed with spermatozoa of 1 patient suggest a normal functionality of sperm flagellum and proximal centriole, even if all cilia structure share common some structural elements and common mechanism of genesis (11, 33).

The functionality of the sperm centriole has been confirmed in patient 3, through the IVF procedure which succeeded in the birth of a healthy child.

The absence of obvious correlation between genotype and phenotype of BBS patients has been already mentioned in a more general context, not focused on reproduction (16). The most frequent mutation, a single missense mutation of exon 12 of *BBS1* (p.M390R) known to induce sometimes a mild phenotype, seems to lead, in our series, to moderate abnormalities of genitalia, suggesting a moderate fetal hypothalamo-pituitary impairment and finally a moderate fetal androgen deficit.

Abnormal tail morphologies of the spermatozoa were observed often in our series (patient 1, patient 3, patient 6, and patient 10) without significant impact on their motility. In addition, the structure of the axoneme in our patients was not specifically disturbed in TEM analysis. These observations contrast with those in mice carrying similar mutations: spermatozoa of *Bbs1* (M390R/M390R) knockout mice presented no flagellum even if the cilia of these mice presented a normal axonemal structure, including 9 peripheral microtubules doublets + 1 central pair arrangement of axonemal microtubules, and elongated cilia with abnormally swollen distal ends suggesting the mutation may impair the completion of flagella assembly (48). Similarly, *Bbs4*-null mice develop normally their somatic motile and primary cilia, suggesting that *Bbs4* is dispensable for global cilia genesis, but interestingly, male *Bbs4-*null mice do not form spermatozoa flagella, suggesting a difference in the formation of the different types of tails (49). Other *Bbs* null mice (*Bbs2, Bbs7*) present also disturbed sperm flagellum synthesis (50, 51). Although *BBS6* belongs to another family of BBS proteins, characterized as chaperonins, *Bbs6*-null mice present also impaired spermatozoa tail synthesis (52). In 2008, Walsh et al highlighted an important role of chaperonin proteins in the fusion mechanism of acrosomal function (acrosomal building by proacrosomal vesicles fusion and acrosomal reaction by acrosome membrane fusion to plasma membrane of sperm head) (53). Since several BBS proteins interact with other chaperonin proteins, it is possible that mutations of BBS proteins of the chaperonin-like family have an impact on sperm acrosomal function and thus on spontaneous fertility. In this case, the ART solution would consist in carrying out an ICSI-IVF (which is what was performed for the patient consulting for infertility in our series).

## Psychology and Implications for Sexual Behavior in BBS

Intellectual disability is a variable feature of BBS and like in earlier reports (54, 55), our patients had Verbal Intellectual Quotient close to 80 (60.5-102; mean and median = 78.5). Nevertheless, all except 1 had a professional activity (from groom to telephone advisor).

Similar to the observation made by Kerr et al (54), we observed emotional immaturity in some BBS patients, with frequent inappropriate emotional outbursts but, in contrast, no disinhibited behavior or inability to recognize social cues.

Disability linked to blindness, frequent lack of self-confidence mainly due to obesity, associated with these psychological traits could explain the low proportion of BBS patients living with a partner (2 out of 11 in our series) and consultation for reproductive assistance (only 1 out of 11).

## Conclusion

BBS is a pleiotropic syndrome affecting reproductive prognostic through: (i) fetal hypogonadotropic hypogonadism resulting in micropenis, short scrotum, and cryptorchidism, (ii) variable impairment in spermatogenesis due to the proximity of testes to abdomen (iii) susceptibility to cystic formation of genital tract leading to partial obstruction of genital ducts, and (iv) sexual/reproductive behavior linked to psychological traits of BBS patients.

However, in contrast with the literature we suggest that: (i) the hypothalamic-pituitary-gonadal axis can function normally in adults despite a frequent severe obesity, (ii) the sperm motility can be normal, and (iii) sperm is able to fertilize mature oocyte and the centriole is able to conduct the first zygote division.

Very few cases of spontaneous fatherhood are reported, maybe more linked to an impaired sexual behaviour. Our study is reassuring about the possibility for male BBS patients to benefit from reproductive medicine care.

## Data Availability

The datasets generated during and/or analyzed during the current study are not publicly available but are available from the corresponding author on reasonable request.

## Abbreviations

ART: assisted reproductive technology
BBS: Bardet-Biedl syndrome
CHH: congenital hypogonadotropic hypogonadism
GnRH: gonadotropin-releasing hormone
ICSI: intracytoplasmic sperm injection
IVF: in vitro fertilization
LH: luteinizing hormone
TEM: Transmission Electron Microscopy
VIQ: verbal intelligence quotient

## Additional Information

***Disclosure Summary:*** This study explores each step of reproduction in a cohort of 11 Bardet-Biedl male patients, from embryology of genitalia to hormonal status, sperm analysis and psychological profile, discussing the hypothetical impact of the ciliopathy.
