# Erratum to: “Reproduction Function in Male Patients With Bardet Biedl Syndrome”

**DOI:** 10.1210/clinem/dgaa897

**Published:** 2020-12-22

In the above-named article by Koscinski I, Mark M, Messaddeq N, Braun J, Celebi C, Muller J, Zinetti-Bertschy A, Goetz N, Dollfus H, and Rossignol S (*J Clin Endocrinol Metab.* 2020;105(12);1–13. doi: 10.1210/clinem/dgaa551), the following typographical error occurred in the published paper:

The order of the first and second name of each author was reversed. The author names should be listed as follows:

Isabelle Koscinski, Manuel Mark, Nadia Messaddeq, Jean Jacques Braun, Catherine Celebi, Jean Muller, Anna Zinetti-Bertschy, Nathalie Goetz, Hélène Dollfus, and Sylvie Rossignol.

The names have been corrected in the publication online.

doi: 10.1210/clinem/dgaa551

